# Genome Sequences of the Guillain-Barré Syndrome Outbreak-Associated *Campylobacter jejuni* Strains ICDCCJ07002 and ICDCCJ07004

**DOI:** 10.1128/genomeA.00256-13

**Published:** 2013-05-23

**Authors:** Maojun Zhang, Xianwei Yang, Hongying Liu, Xiayang Liu, Yufen Huang, Lihua He, Yixin Gu, Jianzhong Zhang

**Affiliations:** State Key Laboratory for Infectious Disease Prevention and Control, National Institute for Communicable Disease Control and Prevention, Chinese Centre for Disease Control and Prevention, Beijing, Chinaa; Beijing Genomics Institute, Tianjin, Chinab; Beijing Genomics Institute, Shenzhen, Chinac

## Abstract

The first world-known and largest outbreak of 36 cases of Guillain-Barré syndrome caused by a preceding *Campylobacter jejuni* infection was reported previously in China. During the outbreak, *Campylobacter jejuni* strain ICDCCJ07002 was isolated from a patient with persistent diarrhea for 21 days, and *C. jejuni* strain ICDCCJ07004 was from a healthy carrier without any clinical symptoms at the same time. Here, we report the draft genome sequence of strain ICDCCJ07002 (1,698,407 bp, with a G+C content of 30.45%) and the genome resequencing result of strain ICDCCJ07004 (1,701,584 bp, with a G+C content of 30.51%), and we compared these with the completed genome of *C. jejuni* strain ICDCCJ07001.

## GENOME ANNOUNCEMENT

In 2007, an outbreak of 36 Guillain-Barré syndrome (GBS) cases was reported in a township in Jilin province, China. Serologic and bacterial studies proved that an antecedent *Campylobacter jejuni* infection triggered this event. Four *C. jejuni* isolates, ICDCCJ07001, ICDCCJ07002, ICDCCJ07003, and ICDCCJ07004, were obtained during the outbreak. The entire isolates had identical types by pulsed-field gel electrophoresis (PFGE) and multilocus sequence typing (MLST), but the serotype of ICDCCJ07004 is different from that of the other three isolates, which have the same penner serotype, HS:41 (1). Besides, ICDCCJ07002 has a different outer core structure on the lipooligosaccharide from that of ICDCCJ07001 (unpublished data). We previously finished the completed genome sequence for ICDCCJ07001 (2). The draft genome sequence for ICDCCJ07002 and the resequencing for ICDCCJ07004 were carried out in this study.

The genome of ICDCCJ07002 was sequenced by a whole-genome shotgun strategy using the Illumina HiSeq 2000 at the Beijing Genomics Institute (BGI) in Shenzhen, China. The genome sequences were assembled *in silico* using SOAP*denovo*, resulting in 14 contigs with an N_50_ length of 243,746 bp. The gene prediction, the functional annotation for the predicted coding sequences (CDSs), and the findings for tRNAs and rRNAs were accomplished as described previously (3). The draft genome of ICDCCJ07002 includes 1,698,407 bases with a G+C content of 30.45% and contains 1,754 CDSs. An estimated 94.0% of nucleotides are predicted CDSs. The 1,303 CDSs annotated by Gene Ontology (GO) can be classified into 20 GO categories, and 1,201 CDSs can be annotated in the KEGG orthology system.

Genome resequencing of ICDCCJ07004 was also performed at BGI using the Illumina HiSeq 2000. The completed genome sequence of ICDCCJ07001 (accession no. NC_014802) was used as the reference genome. The sequences of ICDCCJ07004 were assembled using SOAP*denovo* 1.05 (with parameter *k* = 31). In total, 66 scaffolds and 238 contigs, with a G+C content of 30.51%, were obtained. The sequence coverage and the average depth were 99.85% and 100%, respectively, using the software SOAPaligner 2.21, compared with ICDCCJ07001. The 1,306 CDSs annotated by GO and 1,198 CDSs can be annotated in the KEGG orthology system.

### Nucleotide sequence accession numbers.

This Whole-Genome Shotgun project has been deposited at DDBJ/EMBL/GenBank under the accession no. APNP00000000 for *C. jejuni* ICDCCJ07002 and the accession no. APNQ00000000 for *C. jejuni* ICDCCJ07004.

## References

[1] Zhang M Li Q He L Meng F Gu Y Zheng M Gong Y Wang P Ruan F Zhou L Wu J Chen L Fitzgerald C Zhang J 2010 Association study between an outbreak of Guillain-Barre syndrome in Jilin, China, and preceding *Campylobacter jejuni* infection. Foodborne Pathog. Dis. 7:913–919 20455754 10.1089/fpd.2009.0493

[2] Zhang M He L Li Q Sun H Gu Y You Y Meng F Zhang J 2010 Genomic characterization of the Guillain-Barre syndrome-associated *Campylobacter jejuni* ICDCCJ07001 isolate. PLoS One 5:e15060 21124772 10.1371/journal.pone.0015060 PMC2993937

[3] Lin B Lu G Li S Hu Z Chen H 2012 Draft genome sequence of the novel agarolytic bacterium *Aquimarina agarilytica* ZC1. J. Bacteriol. 194:2769 22535944 10.1128/JB.00311-12 PMC3347208

